# Radiotherapy Advances in Pediatric Neuro-Oncology

**DOI:** 10.3390/bioengineering5040097

**Published:** 2018-11-04

**Authors:** Ethan B. Ludmir, David R. Grosshans, Kristina D. Woodhouse

**Affiliations:** Department of Radiation Oncology, Unit 97, The University of Texas MD Anderson Cancer Center, 1515 Holcombe Blvd, Houston, TX 77030, USA; ebludmir@mdanderson.org (E.B.L.); dgrossha@mdanderson.org (D.R.G.)

**Keywords:** radiotherapy, pediatric oncology, brain tumors, proton beam therapy, intensity-modulated radiotherapy, magnetic resonance imaging, image guidance

## Abstract

Radiation therapy (RT) represents an integral component in the treatment of many pediatric brain tumors. Multiple advances have emerged within pediatric radiation oncology that aim to optimize the therapeutic ratio—improving disease control while limiting RT-related toxicity. These include innovations in treatment planning with magnetic resonance imaging (MRI) simulation, as well as increasingly sophisticated radiation delivery techniques. Advanced RT techniques, including photon-based RT such as intensity-modulated RT (IMRT) and volumetric-modulated arc therapy (VMAT), as well as particle beam therapy and stereotactic RT, have afforded an array of options to dramatically reduce radiation exposure of uninvolved normal tissues while treating target volumes. Along with advances in image guidance of radiation treatments, novel RT approaches are being implemented in ongoing and future prospective clinical trials. As the era of molecular risk stratification unfolds, personalization of radiation dose, target, and technique holds the promise to meaningfully improve outcomes for pediatric neuro-oncology patients.

## 1. Introduction

Cancers of the central nervous system (CNS) are the most common solid tumors among pediatric patients, with an incidence of approximately 3000 new cases annually, accounting for 25% of all pediatric tumors [1]. Over the course of the last several decades, the prognosis for these malignancies has improved steadily, with the most recent nationwide data reporting an estimated 72% 5-year overall survival rate for the pooled cohort of pediatric patients with primary CNS tumors [1]. Improvements in the prognoses of these tumors have been made owing to a combination of factors, including advances in neurosurgery [2,3], diagnostic radiology [3,4,5], radiation oncology [6,7], and medical oncology [8,9], among others. In this review, we will provide an overarching view of recent advances in radiation oncology relevant to pediatric neuro-oncology, as well as look to the future of radiotherapy (RT) in the treatment of these challenging lesions.

## 2. Role of RT in Pediatric Neuro-Oncology

Pediatric brain tumors represent a heterogeneous group of lesions, whose clinical and biological characteristics vary widely; treatment approaches for these lesions can differ markedly between tumor types, patient demographics, and clinical context. Multimodality therapy for these tumors may consist of a combination of maximal safe neurosurgical resection, RT, and systemic chemotherapy or targeted agents. For many benign or low-grade lesions that are accessible surgically, neurosurgical resection alone may afford excellent outcomes, often with acceptable rates of toxicity [10]. On the other hand, for aggressive tumors, combination approaches including surgery, RT, and chemotherapy are indicated to achieve optimal outcomes [11]. 

When RT is indicated, the intent and target can vary depending on a number of factors as well. Among patients with embryonal brain tumors, such as those with medulloblastoma, RT is recommended in the adjuvant setting (following resection), generally targeting the entire brain and spine (craniospinal irradiation, CSI) as well as a boost to the tumor bed [12]. The aim of CSI in this context is to eradicate microscopic disease elsewhere in the CNS, particularly given the proclivity for embryonal tumors to disseminate via cerebrospinal fluid (CSF) pathways [12]. For patients with high-grade unresectable tumors, such as those with diffuse intrinsic pontine glioma (DIPG), RT remains *de facto* the sole therapeutic option available to delay progression and death. Thus, with a range of adjuvant, definitive, and palliative indications, RT plays a significant role in the treatment of most childhood brain tumors.

## 3. Advances in Treatment Planning

For the past two decades, RT treatment planning has relied on the use of computed tomography (CT) simulation. CT simulation allows providers, including the radiation oncologist as well as radiation therapist, to optimize the patient position for subsequent treatments. For patients undergoing RT to the brain, the simulation process often involves the manufacture and customization of a thermoplastic mask to immobilize the head. Following immobilization, axial CT images are acquired, transferred to the treatment planning system, and utilized to determine target volumes. These target volumes, including the gross tumor volume (GTV), clinical tumor volume (CTV), and planning target volume (PTV) are contoured onto the acquired CT simulation images. Also contoured are critical organs-at-risk or avoidance structures, including the brainstem, spinal cord, optic nerves, optic chiasm, cochleae, hippocampi, and others. Through delineation of these structures in the contouring process, treatment planning can proceed to optimize the dose to the target volumes while minimizing dose to critical normal structures.

One pitfall of this process in the treatment of brain tumors, however, is the relatively poor soft-tissue contrast associated with CT imaging, particularly in the neuro-axis. Therefore, from the initial development of CT simulation, efforts were made to incorporate the high degree of soft-tissue contrast with magnetic resonance imaging (MRI) into the treatment planning process [13,14,15]. To accomplish this, MRI sequences (generally from diagnostic scans) are co-registered to the CT simulation images. The co-registration, or ‘fusion’, of MRI series to CT imaging relies on transformation models to align these scans to one another, which can be optimized to account for organ distortion [16,17,18]. Multiple studies have demonstrated that the addition of MRI co-registration for treatment of brain tumors has resulted in substantial changes to both the contoured target volumes as well as organs-at-risk [15,19]. Variations in tumor delineation during treatment planning in the absence of co-registered MRI sequences carries significant clinical consequences, with the potential for under-treatment of disease as well as over-treatment of critical normal structures. 

**Figure 1:**
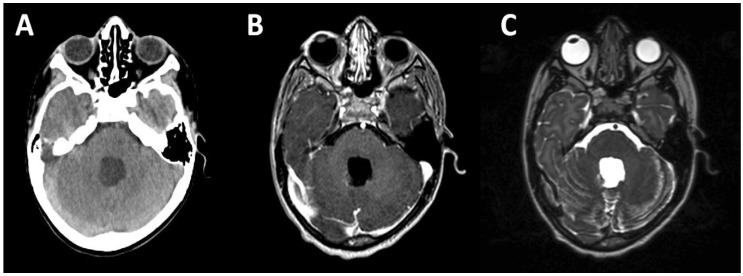
CT and MRI simulation. Axial images from CT (**A**) and MRI (**B**,**C**) simulation for radiation treatment planning purposes. This patient is an 8-year-old male who presents for adjuvant radiotherapy three weeks following gross total resection of average-risk posterior fossa medulloblastoma. CT simulator axial image showing the resection cavity is shown in (**A)**; (**B**,**C**) show MRI simulator axial images from the same plane; T1 post-gadolinium contrast sequence is shown in (**B**), and the T2 sequence is shown in (**C**). The MRI simulator allows for identical positioning and immobilization to be achieved, facilitating fusion of CT and MRI images as demonstrated here.

Given the centrality of MRI in treatment planning for brain tumors (among others), an increasing number of institutions are pursuing the use of dedicated MRI simulators [16,19]. These MRI simulators allow for MRI sequences to be obtained with appropriate patient positioning using requisite immobilization devices for treatment planning purposes (Figure 1). Whereas co-registered MRI data from diagnostic imaging are derived from scans in which patients are not in the treatment position or with immobilization devices such as a mask, dedicated MRI simulators allow for high-fidelity MRI sequences to be obtained in the treatment position [20]. This focus on geometric consistency between MRI acquisition and subsequent RT treatments facilitates utilization of MRI data for contour definition (Figure 1) [20,21]. Clinically, this approach is particularly advantageous in the context of adjacent target volumes and critical organs-at-risk, for instance treatment of posterior fossa tumors adjacent to brainstem, or suprasellar/sellar tumors adjacent to optic pathway structures; in these cases, accurate delineation of target versus non-target structures is paramount to avoid severe toxicity such as brainstem necrosis or visual impairment, respectively. 

Furthermore, efforts to date utilizing MRI co-registration or MRI simulation have generally used these as an adjunct to CT simulation. CT simulation provides a spatial electron density map (via CT-derived Hounsfield units), which is then used to model conformal RT treatment plans [22]. Modeling dose distribution accurately during treatment planning is contingent on this electron density map. Looking ahead, ongoing studies are attempting to utilize MRI sequences to infer electron density, so that accurate treatment planning could proceed with MRI simulation alone [16,21,23,24]. This would be particularly helpful for pediatric patients, in whom limiting radiation exposure (i.e., through reducing the number of CT scans performed) is of biological and clinical importance [25]. Logistically, pediatric patients up to the age of eight years old often require sedation to tolerate both simulation and radiation treatments; consolidating simulation to one scan (an MRI) may similarly provide practical advantages for this special patient population. 

Specific MRI sequences and techniques also hold promise to enhance RT planning and delivery. Conventionally, commonly utilized MRI sequences to anatomically define target volumes as well as normal structures include T1 post-gadolinium-contrast and T2 fluid-attenuated inversion recovery (FLAIR) [16,26,27,28]. While T1 post-contrast and T2 FLAIR sequences yield excellent intracranial soft tissue contrast for defining volumes (T2 FLAIR being particularly helpful for assessing tissue peritumoral edema and gliosis), functional MRI sequences may expand the role of MRI in treatment planning as well as clinical outcomes [21,26]. Diffusion-weighted MRI sequences (also known as diffusion-weighted MR imaging, or DWI), for instance, provide an assessment of water diffusion through tissue, which can assist in assessing cellularity of a particular voxel [21]. Increased water diffusion distance reflects decreased cellularity—an indicator of treatment response. Supporting this, DWI mid-way through the course of RT has been shown among brain tumor patients to provide a radiographic biomarker of treatment response and subsequent long-term survival [29,30,31]. MR spectroscopy may provide further functional and metabolic data, using tissue metabolite nuclei characteristics to assess the chemical composition of a given voxel. With expertise and support from experienced radiologists, MR spectroscopy can yield a voxel-by-voxel map of data related to cell turnover, hypoxia, and other physiologic parameters that can inform treatment planning and response [21,26,32,33,34]. While promising, these functional imaging techniques are only now beginning to be incorporated into clinical practice. 

## 4. Advances in RT Delivery

### 4.1. Advanced Photon RT Techniques

With the integration of CT simulation and three-dimensional treatment planning over the prior decades, photon-based RT techniques have similarly progressed. Moving beyond the era of two-dimensional (2D) and three-dimensional conformal RT (3D-CRT), intensity-modulated RT (IMRT) has entered mainstream clinical practice and use, including among pediatric neuro-oncology patients. The underlying premise for IMRT is rooted in the use of inverse-planning systems that rely on objective functions to optimize a treatment plan. Based on pre-specified target volume goals and organ-at-risk constraints, inverse-planning algorithms can generate plans using multiple beam arrangements (i.e., 8 or 9 beams) with non-uniform beam fluences to optimize the objective functions. These plans generally rely on the use of multileaf collimators (often made of a high-atomic-number [high-Z] materials such as tungsten) that subdivide each beam into several small ‘beamlets’, each of which in turn can have variable fluences to achieve the desired objective functions. Collectively, this approach can yield highly conformal plans in which high-dose regions can be shifted away from critical structures, though usually with a lower-dose ‘bath’ to a larger region of uninvolved tissue. 

In the treatment of brain tumors, IMRT has been shown to improve target conformity as well as critical structure sparing when compared to standard 3D-CRT approaches [35,36,37]. These dosimetric advantages to IMRT are supported by clinical data, which demonstrate comparable rates of disease control with IMRT approaches [38,39,40], but decreased RT-related toxicity, particularly ototoxicity [37,41,42]. Expanding on IMRT, arc-based therapies have increasingly been utilized. These approaches, such as volumetric-modulated arc therapy (VMAT), rely on coplanar intensity-modulated arcs in which the linear accelerator gantry rotates around the patient [43,44]. As the gantry rotates around the patient, dynamic modulation can occur for the speed of gantry rotation, the beam-shaping aperture (including multi-leaf collimator), as well as the dose delivery rate [43,44,45,46]. The result is a highly-conformal treatment plan, comparable and often superior to IMRT with regard to target volume coverage, coverage homogeneity, and normal tissue sparing [44,45,47,48]. However, the primary advantage of arc-based techniques is the speed of treatment delivery [44], in which a single arc can be delivered over a few (2–3) min. Given that most VMAT plans rely on one to three arcs, the total time for patients on the treatment table may be reduced from approximately 30 min with IMRT to 10–15 min with VMAT, in our experience. This difference is particularly advantageous for pediatric patients, whose tolerance for treatments may be more limited than adult patients, and many of whom may require sedation while undergoing treatment. A caveat to the above is that the low-dose ‘bath’ may be more pronounced with VMAT, and certain clinical and dosimetric instances may arise where 3D-CRT approaches may provide optimal sparing of a specific at-risk structure [45,48].

### 4.2. Particle Therapy

In conjunction with advanced photon-based RT, particle therapy, including proton beam therapy (PBT), has now entered mainstream clinical practice among pediatric neuro-oncology patients [49,50,51]. The underlying principle of particle therapy, with PBT or heavier ions such as carbon ions, relies on the physical properties of particle beam dose deposition. With photon beams, radiation is deposited from its entrance into the body to a maximum dose, and then continues to deposit dose as it exits through to the other side of the body. The maximum depth dose for clinically-utilized photon beams (with energies between 4 MV and 18 MV) ranges between approximately 1.5 cm and 3.5 cm. Beyond these maximum depths, photon beams continue to deposit dose until exiting the other side of the body and striking a shielded wall on the opposite side of the treatment room. In contrast, proton beams (and more particle beams more generally) decrease velocity as they pass through tissue, depositing more energy with decreasing velocity until reaching a stopping depth in which the bulk of their energy is deposited. Distal to this characteristic depth, limited further energy is deposited in tissue, resulting in essentially no exit dose past the maximum depth dose for a proton beam. The characteristic peak energy deposition at a given depth is known as the Bragg curve (or Bragg peak). The specific depth of a Bragg peak for a mono-energetic proton beam is dependent on the energy of the proton beam (usually ranging between 70 and 250 MeV). By utilizing poly-energetic proton beams in clinical use, the Bragg peak becomes “spread-out” such that a target volume can be treated across its thickness with a proton beam; even with a poly-energetic proton beam, however, the dose drop-off at the end of the peak remains, with no dose beyond the distal edge of the spread-out Bragg peak. The primary advantage of PBT, therefore, is the ability to deliver RT with minimal exit dose. 

**Figure 2:**
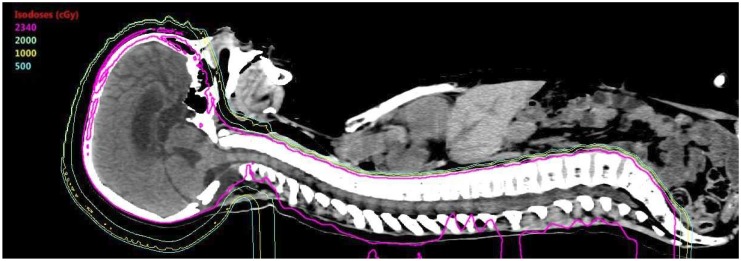
Proton beam therapy CSI. Representative mid-sagittal image of an 11-year-old male patient status post gross total resection for average-risk posterior-fossa medulloblastoma being planned for adjuvant radiotherapy (RT) including craniospinal irradiation (CSI). Shown here are isodose lines (colored lines) for the radiation treatment plan for the CSI component of this patient’s adjuvant RT. CSI fields cover the complete brain and spinal cord to the termination of the thecal sac in the sacrum. The 100% isodose line (23.4Gy[RBE]) covers the entire target volume as shown in this image. As this patient is being treated with PBT, the posterior-positioned spinal fields have their terminal Bragg peak at the anterior-most portion of the vertebral bodies. Therefore, there is no exit dose anterior to the vertebrae, as shown with the isodose lines, including the low-dose 5Gy[RBE] isodose line (cyan). No dose is seen treating anterior structures such as the heart, liver, thyroid, gastrointestinal organs, or other anterior structures seen in this representative sagittal slice.

Given the susceptibility of children to late effects from RT (including neurocognitive deficits, neuroendocrine deficits, audiovisual toxicity, growth abnormalities, and second malignancies, among others), the potential for PBT to minimize normal tissue radiation exposure through the absence of exit dose represents an enticing opportunity to enhance the therapeutic ratio. Multiple dosimetric studies have demonstrated a significant reduction in dose to critical organs at risk, as well as uninvolved brain more generally, in the treatment of diverse pediatric brain tumors using PBT; these studies confirmed dosimetric benefits of PBT over advanced photon-based techniques (including IMRT) for patients with medulloblastoma, ependymoma, and craniopharyngioma [52,53,54,55,56,57]. This has been most pronounced for patients who require CSI, such as medulloblastoma patients (Figure 2). With CSI, posterior positioning of proton beam(s) targeting the spinal canal as part of CSI allows for dose to cover the spinal canal and vertebral bodies, with minimal dose anteriorly into critical organs including thyroid, heart, lungs, gastrointestinal structures (i.e., pancreas), and ovaries, among others (Figure 2) [52,53,57,58]. So pronounced are the dosimetric differences in sparing anterior structures with PBT for CSI that the pediatric radiation oncology community has debated in recent years whether PBT represents the only standard of care option for pediatric patients requiring CSI (Figure 2) [59,60]. 

Dosimetric data have been supported by clinical data for PBT thus far. Across multiple disease sites, reviewed individually elsewhere, PBT has provided disease control rates comparable to those with photon-based RT, while often providing clinically-meaningful reductions in toxicity [7,49,50,51,58,61,62,63,64,65,66]. Furthermore, PBT does not appear to increase the risk of second malignancies among treated patients as compared with photon RT [67,68]. The possibility of increased second malignancy risk with PBT arose due to concerns regarding increased secondary neutron production with PBT, particularly with the use of patient-specific brass apertures and scattering devices utilized with certain PBT treatment plans [69,70]. Such secondary neutrons might, hypothetically, increase whole-body non-target radiation exposure and increase second malignancy risk [70], a relevant concern for pediatric patients whose baseline risk for RT-related second malignancy is higher than that of adults. However, both modeling data as well as clinical evidence to date suggests that if anything PBT may decrease second malignancy, likely owing to the absence of low-dose ‘bath’ as occurs with advanced photon-based techniques such as IMRT or VMAT [49,67,68,71]. 

The underlying technology and treatment delivery systems for PBT continue to progress as well. Earlier iterations of PBT have relied on the use of passive-scatter PBT (PSPT). PSPT utilizes a range modulator wheel to generate a spread-out Bragg peak from an initial monoenergetic proton beam; the proton beam is then shaped with brass apertures to sharpen the lateral border of the proton beam as well as compensator to modulate the distal edge of the beam. PSPT is somewhat limited with regard to modulating dose for structures proximal to the target volume in the path of the beam, and similarly may not provide conformal treatment plans for irregularly-shaped targets. Scanning-beam PBT, as opposed to PSPT, relies on proton ‘beamlets’ of discrete energies which can be delivered in layers (with each layer/depth reflecting a specific beamlet energy). Rather than using a range modulator, scanning-beam PBT allows for ‘dose painting’ of the target volume with these beamlets, and can afford high conformality of irregularly-shaped targets. Scanning-beam PBT therefore allows for intensity-modulated proton therapy (IMPT) to be possible, wherein ‘dose painting’ of a target volume can achieve highly-conformal dose distributions surpassing both advanced photon-based techniques as well as PSPT [72,73]. Emerging clinical data highlight high rates of disease control with reduced toxicity utilizing IMPT, primarily in the treatment of head and neck malignancies [74,75,76]. These advances in PBT technology, as well as challenges with regard to uncertainties with PBT, have been reviewed elsewhere and are beyond the scope of this review [49,51]. However, ongoing efforts to understand the radiobiological differences between proton and photon beams are increasingly revealing the complexities of particle therapy, as well as the enhanced biological effectiveness of protons as compared with photons [49]. The higher relative biological effectiveness of protons carries both potential advantages with regard to tumor cell killing, as well as risks in terms of increased risk of toxicity to critical structures that receive dose (such as the brainstem in the case of posterior fossa tumors) [49,77]. 

Heavier ion particle therapy, such as carbon ion radiotherapy, has also emerged as a treatment option for pediatric patients. Carbon ion therapy, as with PBT, demonstrates a Bragg peak dose distribution with minimal exit dose; however, the biological effectiveness of heavier ions is thought to be greater than that of both photons and protons [78]. These properties suggest that heavy ion therapy may be particularly helpful for radioresistant tumors, such as osteosarcoma (the most common pediatric bone tumor). Early data utilizing definitive heavy ion therapy for unresectable pediatric osteosarcoma are promising [79,80,81], and future studies exploring the role of heavy ion therapy in the treatment of pediatric patients are underway.

### 4.3. Stereotactic Approaches

Stereotactic RT represents another option emerging within pediatric radiation oncology. Stereotactic approaches are advanced photon-based techniques that are highly-focal and precise, delivering large fractional doses of radiation largely to small, spherical targets. Stereotactic RT is often delivered as a single fraction (generally called stereotactic radiosurgery [SRS] if delivered as a single treatment) or over a few fractions (usually five or fewer treatments, called stereotactic radiotherapy [SRT]). SRS and SRT can be delivered using a number of systems, including the Gamma Knife system (in which a patient sits inside a ‘helmet’ with multiple non-coplanar slits, which in turn allow for dose to be delivered from cobalt-60 sources to an intracranial target) as well as modified linear accelerators (including dedicated radiosurgery liner accelerators such as the CyberKnife system) [6]. The ideal target for such stereotactic approaches is small and spherical, and consequently this approach has been largely utilized for patients (primarily adults) with brain metastases. In the pediatric setting, stereotactic techniques present unique opportunities as well as challenges. While the proportion of pediatric patients with brain metastases is marginal as compared with that of adult patients, pediatric patients with both neoplastic as well as certain vascular lesions may benefit from SRS/SRT. Since SRS and SRT are delivered in one or a few fractions, respectively, this significantly reduces the burden of prolonged treatment duration on pediatric patients. On the other hand, as SRS and SRT approaches often require more invasive immobilization methods, such as the use of a stereotactic head ‘frame’ which is affixed to the skull with the use of metal pins, pediatric patients may be more likely to require sedation with the procedure. However, if the treatment is delivered only once (i.e., SRS) over a single day, this invasive but shorter approach may represent an advantage compared with daily sedation for a conventional RT course that would otherwise require 30 treatments over six weeks, for instance. 

**Figure 3:**
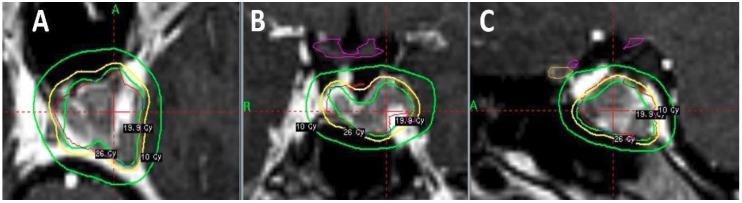
Stereotactic RT in the pediatric setting. This case is that of a 17-year-old female who presents with enlarging residual pituitary adenoma. The patient was initially treated one year prior with subtotal trans-nasal endoscopic resection of a non-secreting pituitary adenoma. On subsequent MRI surveillance, she was noted to have slow enlargement of residual pituitary adenoma. She was therefore recommended for salvage stereotactic RT to this residual disease. She was treated with a single-fraction of stereotactic radiosurgery, prescribing 19.9 Gy to the 50% isodose line. Shown here are representative (**A**) axial; (**B**) coronal; and (**C**) sagittal sections of the plan, with 10 Gy, 19.9 Gy, and 26 Gy isodose lines. Critical structures are appropriately avoided using this highly-conformal single-treatment technique, including optic chiasm (purple contour) and left optic nerve (orange contour).

SRS and SRT themselves have been utilized heterogeneously among pediatric patients. This includes efforts to incorporate SRS as a ‘boost’ for high-risk ependymoma patients being treated with adjuvant RT [6,82,83], as definitive treatment for low-grade gliomas [84,85,86], or in the setting of recurrent/residual disease, often within previously-irradiated tissue [6,83,87,88,89,90,91,92]. One such case of utilizing SRS to treat a recurrent pituitary adenoma in a pediatric patient is highlighted in Figure 3. SRS has similarly been shown to represent a safe and effective treatment option for pediatric arteriovenous malformations, discussed and reviewed extensively elsewhere [93]. Beyond this, stereotactic approaches have been considered in a variety of clinical contexts (often borrowing upon experiences treating adult patients). Single-fraction high-dose palliative approaches with stereotactic RT are promising options, as is the use of stereotactic RT for the ablative treatment of oligometastatic disease [94,95]. Reirradiation may be another context where SRS/SRT may be helpful, given the rapid dose fall-off with such treatment plans and consequent minimal reirradiation of critical normal structures being exposed to another course of RT [96]. Stereotactic fractionated approaches are similarly considered for higher dose-per-fraction boost fields among certain pediatric ependymoma protocols [97]. While the indications for SRS and SRT among pediatric neuro-oncology patients remain heterogeneous, they represent an emerging technique for the treatment of intracranial lesions across diverse clinical contexts.

### 4.4. Improving Image Guidance

Central to the use of advanced RT delivery technologies is improved patient alignment confirmation. For highly-conformal RT techniques, including IMRT, VMAT, PBT, and stereotactic RT, patient set-up errors and positional deviations can dramatically impact the treatment plan, potentially causing target volumes to be under-treated and normal structures to be over-treated. Such errors can therefore result in decreased tumor control, increased treatment-related toxicity, or both. Therefore, to support advanced RT delivery technologies, advanced alignment verification technologies have emerged. At the heart of these alignment verification methods are techniques known as image-guided radiation therapy (IGRT). IGRT most commonly includes techniques such as (1) kilovoltage (kV) planar radiographs from X-ray devices mounted in the treatment room (often mounted as part of the treatment linear accelerator/gantry itself), or (2) volumetric imaging using cone beam computed tomography (CBCT) scans, which are similarly incorporated into the treatment room [98,99]. kV-IGRT provides physicians with planar radiographs, often obtained in two radiographic planes (for instance, one anteroposterior radiograph and one lateral radiograph), that facilitate accurate alignment to bony landmarks [98]. CBCT, on the other hand, allows for three-dimensional volumetric imaging with CT-level soft tissue resolution [98]. For most intracranial tumors, our institutional practice, consistent with many other academic centers [98], has been to utilize daily kV-IGRT to ensure accurate bony alignment. Given the reliability of bony landmarks for intracranial target volumes, whose positions are markedly more consistent than targets in the abdomen or pelvis, for instance, daily kV imaging has been widely adopted as an effective IGRT tool for advanced RT techniques in the treatment of intracranial tumors [98,99]. Some institutions have more routinely employed CBCT imaging for pediatric neuro-oncology patients, suggesting that the set-up uncertainty margin added to the CTV to generate the PTV can be reduced with the use of CBCT [100]. However, concerns regarding radiation exposure to pediatric patients undergoing CBCT IGRT have been raised [25,99]. With doses of approximately 3 cGy per CBCT [101], frequent CBCT imaging (daily or weekly) has the potential to substantially increase risk of mutagenesis and second malignancy [25,99]. This additional radiation exposure may also translate into higher rates of infertility and endocrine dysfunction [25,99]. Therefore, our overarching approach, consistent with most high-volume pediatric centers, has been to utilize kV-IGRT to optimize patient alignment and reduce PTV margins; CBCT, however, is used sparingly.

Along these lines, an important caveat is that IGRT as well as adaptive re-planning have been increasingly utilized for target volumes whose size and shape may vary over the treatment course. Perhaps the clearest example of such a tumor within pediatric neuro-oncology is craniopharyngioma. These lesions, comprised of both solid and cystic components, have a propensity to develop dynamic changes in cyst size and position during a course of RT [102,103]. Therefore, adaptive re-planning with repeat imaging has been recommended by multiple groups in the treatment of craniopharyngioma [55,62,102,103,104]. Our practice is generally to have craniopharyngioma patients undergo MRI simulation up-front as well as every one to two weeks while on treatment. Using the MRI simulator, we can obtain MRI imaging to assess cyst dynamics and complete an adaptive radiation plan if needed utilizing these MR images in the treatment position. This paradigm also avoids repeat CT imaging and associated radiation exposure, ideal for pediatric patients as previously discussed.

## 5. Clinical Efforts—Refining Target Volumes

### 5.1. Shrinking Field Sizes

Cooperative group clinical studies have further advanced pediatric neuro-oncology with efforts to refine target volumes. By decreasing field sizes, many of these trials aim to reduce RT-related toxicity and exposure without compromising disease control. For medulloblastoma patients, institutional data suggested that the standard boost field encompassing the entire posterior fossa (PF) may be excessive, and that rather a smaller ‘involved field’ (IF) boost volume targeting the tumor bed (with margin) would be sufficient [105,106]. To test whether such a reduced IF boost would provide comparable disease control rates as a PF boost, the Children’s Oncology Group (COG) ACNS 0331 trial randomized standard-risk medulloblastoma patients to IF versus PF boost fields; presented in abstract form, the data demonstrate comparable 5-year event free survival (EFS) between the IF and PF boost arms (82.2% vs. 80.8%) [107]. Consequently, the standard of care has now shifted supporting use of IF boost fields rather than PF boost fields for medulloblastoma patients. Along similar lines, intracranial germ cell tumor (GCT) studies have aimed to reduce field sizes. For intracranial pure germinomas, a highly radiosensitive tumor, efforts sought to reduce definitive RT volumes from CSI to limited-field RT targeting the local tumor alone [108,109]. These prospective European studies demonstrated that such drastic target volume reductions yielded unacceptably high rates of periventricular disease relapse [108,109]. These data, coupled with a large literature review, supported the use of whole-ventricular irradiation (WVI), an intermediate between CSI and limited-field RT [108,109,110]. WVI, while still somewhat larger than limited-field RT targeting the tumor alone, represents a significant reduction in the treatment volume as compared with CSI, and has emerged as the standard for intracranial germinoma, utilized in the most recent COG protocol (ACNS 1123) [111]. For non-germinomatous intracranial germ cell tumors (NGGCTs), which have a higher risk of relapse and a poorer prognosis as compared with pure germinomas, the North American treatment paradigm has centered on the use of induction chemotherapy, with or without second-look surgery, and subsequent CSI [112]. As with medulloblastoma, institutional data suggest that reduced field size to WVI may not compromise disease control [113]; the NGGCT stratum of ACNS 1123 therefore is testing whether WVI, rather than CSI, may be an effective option for those who radiographically respond to induction chemotherapy. 

### 5.2. Optimizing Dose

In conjunction with prospective studies designed to reduce field size, pediatric neuro-oncology trials have also focused on better elucidating the optimal RT doses for patients. These protocols have broadly asked to what extent RT dose can be reduced without compromising clinical outcomes. For standard-risk medulloblastoma patients, for instance, prospective data demonstrated that adjuvant RT with dose-reduced CSI (from 36 Gy to 23.4 Gy) did not result in inferior outcomes, provided patients also received adjuvant chemotherapy following RT [114]. COG ACNS 0331 attempted to further reduce the CSI dose to 18 Gy for standard-risk medulloblastoma patients, based on promising pilot data [107,115]. However, despite these pilot studies, reduced dose CSI from 23.4 Gy to 18 Gy in ACNS 0331 resulted in inferior EFS and overall survival [107]. Therefore, these trials have established 23.4 Gy, and not lower, as the ideal CSI dose for standard-risk medulloblastoma patients [107]. Efforts to determine the appropriate RT dose for pediatric patients are increasingly incorporating radiographic and molecular data to better risk-stratify patients and tailor dose accordingly. For intracranial germinoma patients, ACNS 1123 is assessing the feasibility of dose reduction based on radiographic response to induction chemotherapy [111]. For medulloblastoma patients, ongoing trials in both North America (SJMB12) and Europe (PNET5) are utilizing medulloblastoma molecular risk stratification to tailor RT dose, including dose reduction for lower-risk patients as well as dose intensification for higher-risk patients [116].

## 6. Conclusions

The treatment of pediatric brain tumors with RT continues to evolve with ongoing advances. New technologies, from MRI simulation to particle beam therapy and stereotactic RT, aim to improve the therapeutic ratio further—optimizing disease control and minimizing treatment-related toxicity. As the era of molecular risk stratification unfolds, personalization of radiation dose, target, and technique holds the promise to better the lives of all pediatric neuro-oncology patients.

## References

[1] Ward E., DeSantis C., Robbins A., Kohler B., Jemal A. (2014). Childhood and adolescent cancer statistics, 2014. CA A Cancer J. Clin..

[2] Governale L.S. (2015). Minimally invasive pediatric neurosurgery. Pediatr. Neurol..

[3] Zebian B., Vergani F., Lavrador J.P., Mukherjee S., Kitchen W.J., Stagno V., Chamilos C., Pettorini B., Mallucci C. (2017). Recent technological advances in pediatric brain tumor surgery. CNS Oncol..

[4] Choudhri A.F., Siddiqui A., Klimo P., Boop F.A. (2015). Intraoperative MRI in pediatric brain tumors. Pediatr. Radiol..

[5] Choudhri A.F., Siddiqui A., Klimo P. (2016). Pediatric Cerebellar Tumors: Emerging Imaging Techniques and Advances in Understanding of Genetic Features. Magn. Reson. Imaging Clin. N. Am..

[6] Murphy E.S., Chao S.T., Angelov L., Vogelbaum M.A., Barnett G., Jung E., Recinos V.R., Mohammadi A., Suh J.H. (2016). Radiosurgery for Pediatric Brain Tumors. Pediatr. Blood Cancer.

[7] Eaton B.R., Yock T. (2014). The use of proton therapy in the treatment of benign or low-grade pediatric brain tumors. Cancer J..

[8] Gajjar A., Pfister S.M., Taylor M.D., Gilbertson R.J. (2014). Molecular insights into pediatric brain tumors have the potential to transform therapy. Clin. Cancer Res. Off. J. Am. Assoc. Cancer Res..

[9] Kieran M.W. (2014). Targeting BRAF in pediatric brain tumors. Am. Soc. Clin. Oncol. Educ. Book.

[10] Watson G.A., Kadota R.P., Wisoff J.H. (2001). Multidisciplinary management of pediatric low-grade gliomas. Semin. Radiat. Oncol..

[11] Paulino A.C. (2002). Current multimodality management of medulloblastoma. Curr. Prob. Cancer.

[12] McGovern S.L., Grosshans D., Mahajan A. (2014). Embryonal brain tumors. Cancer J..

[13] Just M., Rosler H.P., Higer H.P., Kutzner J., Thelen M. (1991). MRI-assisted radiation therapy planning of brain tumors--clinical experiences in 17 patients. Magn. Reson. Imaging.

[14] Ten Haken R.K., Thornton A.F., Sandler H.M., LaVigne M.L., Quint D.J., Fraass B.A., Kessler M.L., McShan D.L. (1992). A quantitative assessment of the addition of MRI to CT-based, 3-D treatment planning of brain tumors. Radiother. Oncol. J. Eur. Soc. Ther. Radiol. Oncol..

[15] Thornton A.F., Sandler H.M., Ten Haken R.K., McShan D.L., Fraass B.A., La Vigne M.L., Yanke B.R. (1992). The clinical utility of magnetic resonance imaging in 3-dimensional treatment planning of brain neoplasms. Int. J. Radiat. Oncol. Biol. Phys..

[16] Devic S. (2012). MRI simulation for radiotherapy treatment planning. Med. Phys..

[17] Brock K.K. (2010). Results of a multi-institution deformable registration accuracy study (MIDRAS). Int. J. Radiat. Oncol. Biol. Phys..

[18] Wu X., Dibiase S.J., Gullapalli R., Yu C.X. (2004). Deformable image registration for the use of magnetic resonance spectroscopy in prostate treatment planning. Int. J. Radiat. Oncol. Biol. Phys..

[19] Weber D.C., Wang H., Albrecht S., Ozsahin M., Tkachuk E., Rouzaud M., Nouet P., Dipasquale G. (2008). Open low-field magnetic resonance imaging for target definition, dose calculations and set-up verification during three-dimensional CRT for glioblastoma multiforme. Clin. Oncol. (R. Coll. Radiol.).

[20] Rai R., Kumar S., Batumalai V., Elwadia D., Ohanessian L., Juresic E., Cassapi L., Vinod S.K., Holloway L., Keall P.J. (2017). The integration of MRI in radiation therapy: Collaboration of radiographers and radiation therapists. J. Med. Radiat. Sci..

[21] Metcalfe P., Liney G.P., Holloway L., Walker A., Barton M., Delaney G.P., Vinod S., Tome W. (2013). The potential for an enhanced role for MRI in radiation-therapy treatment planning. Technol. Cancer Res. Treat..

[22] Matsufuji N., Tomura H., Futami Y., Yamashita H., Higashi A., Minohara S., Endo M., Kanai T. (1998). Relationship between CT number and electron density, scatter angle and nuclear reaction for hadron-therapy treatment planning. Phys. Med. Biol..

[23] Scheffler K., Lehnhardt S. (2003). Principles and applications of balanced SSFP techniques. European radiology.

[24] Haase A., Frahm J., Matthaei D., Hanicke W., Merboldt K.D. (2011). FLASH imaging: Rapid NMR imaging using low flip-angle pulses. 1986. J. Magn. Reson..

[25] Brenner D.J., Hall E.J. (2007). Computed tomography--an increasing source of radiation exposure. N. Engl. J. Med..

[26] Liney G.P., Moerland M.A. (2014). Magnetic resonance imaging acquisition techniques for radiotherapy planning. Semin. Radiat. Oncol..

[27] Mazzara G.P., Velthuizen R.P., Pearlman J.L., Greenberg H.M., Wagner H. (2004). Brain tumor target volume determination for radiation treatment planning through automated MRI segmentation. Int. J. Radiat. Oncol. Biol. Phys..

[28] Schad L.R., Bluml S., Hawighorst H., Wenz F., Lorenz W.J. (1994). Radiosurgical treatment planning of brain metastases based on a fast, three-dimensional MR imaging technique. Magn. Reson. Imaging.

[29] Mardor Y., Pfeffer R., Spiegelmann R., Roth Y., Maier S.E., Nissim O., Berger R., Glicksman A., Baram J., Orenstein A. (2003). Early detection of response to radiation therapy in patients with brain malignancies using conventional and high b-value diffusion-weighted magnetic resonance imaging. J. Clin. Oncol..

[30] Hamstra D.A., Galban C.J., Meyer C.R., Johnson T.D., Sundgren P.C., Tsien C., Lawrence T.S., Junck L., Ross D.J., Rehemtulla A. (2008). Functional diffusion map as an early imaging biomarker for high-grade glioma: Correlation with conventional radiologic response and overall survival. J. Clin. Oncol..

[31] Goldman M., Boxerman J.L., Rogg J.M., Noren G. (2006). Utility of apparent diffusion coefficient in predicting the outcome of Gamma Knife-treated brain metastases prior to changes in tumor volume: A preliminary study. J. Neurosurg..

[32] Hoskin P.J., Carnell D.M., Taylor N.J., Smith R.E., Stirling J.J., Daley F.M., Saunders M.I., Bentzen S.M., Collins D.J., d’Arcy J.A. (2007). Hypoxia in Prostate Cancer: Correlation of BOLD-MRI With Pimonidazole Immunohistochemistry—Initial Observations. Int. J. Radiat. Oncol. Biol. Phys..

[33] Payne G.S., Leach M.O. (2006). Applications of magnetic resonance spectroscopy in radiotherapy treatment planning. Br. J. Radiol..

[34] Chang J., Thakur S., Perera G., Kowalski A., Huang W., Karimi S., Hunt M., Koutcher J., Fuks Z., Amols H. (2006). Image-fusion of MR spectroscopic images for treatment planning of gliomas. Med. Phys..

[35] Hermanto U., Frija E.K., Lii M.J., Chang E.L., Mahajan A., Woo S.Y. (2007). Intensity-modulated radiotherapy (IMRT) and conventional three-dimensional conformal radiotherapy for high-grade gliomas: Does IMRT increase the integral dose to normal brain?. Int. J. Radiat. Oncol. Biol. Phys..

[36] Beltran C., Naik M., Merchant T.E. (2010). Dosimetric effect of setup motion and target volume margin reduction in pediatric ependymoma. Radiother. Oncol. J. Eur. Soc. Ther. Radiol. Oncol..

[37] Huang E., Teh B.S., Strother D.R., Davis Q.G., Chiu J.K., Lu H.H., Carpenter L.S., Mai W.Y., Chintagumpala M.M., South M. (2002). Intensity-modulated radiation therapy for pediatric medulloblastoma: Early report on the reduction of ototoxicity. Int. J. Radiat. Oncol. Biol. Phys..

[38] Paulino A.C., Mazloom A., Terashima K., Su J., Adesina A.M., Okcu M.F., Teh B.S., Chintagumpala M. (2013). Intensity-modulated radiotherapy (IMRT) in pediatric low-grade glioma. Cancer.

[39] Polkinghorn W.R., Dunkel I.J., Souweidane M.M., Khakoo Y., Lyden D.C., Gilheeney S.W., Becher O.J., Budnick A.S., Wolden S.L. (2011). Disease control and ototoxicity using intensity-modulated radiation therapy tumor-bed boost for medulloblastoma. Int. J. Radiat. Oncol. Biol. Phys..

[40] Greenfield B.J., Okcu M.F., Baxter P.A., Chintagumpala M., Teh B.S., Dauser R.C., Su J., Desai S.S., Paulino A.C. (2015). Long-term disease control and toxicity outcomes following surgery and intensity modulated radiation therapy (IMRT) in pediatric craniopharyngioma. Radiother. Oncol. J. Eur. Soc. Ther. Radiol. Oncol..

[41] Nanda R.H., Ganju R.G., Schreibmann E., Chen Z., Zhang C., Jegadeesh N., Cassidy R., Deng C., Eaton B.R., Esiashvili N. (2017). Correlation of Acute and Late Brainstem Toxicities With Dose-Volume Data for Pediatric Patients With Posterior Fossa Malignancies. Int. J. Radiat. Oncol. Biol. Phys..

[42] Jain N., Krull K.R., Brouwers P., Chintagumpala M.M., Woo S.Y. (2008). Neuropsychological outcome following intensity-modulated radiation therapy for pediatric medulloblastoma. Pediatr. Blood Cancer.

[43] Otto K. (2008). Volumetric modulated arc therapy: IMRT in a single gantry arc. Med. Phys..

[44] Fogliata A., Clivio A., Nicolini G., Vanetti E., Cozzi L. (2008). Intensity modulation with photons for benign intracranial tumours: A planning comparison of volumetric single arc, helical arc and fixed gantry techniques. Radiother. Oncol. J. Eur. Soc. Ther. Radiol. Oncol..

[45] Wagner D., Christiansen H., Wolff H., Vorwerk H. (2009). Radiotherapy of malignant gliomas: Comparison of volumetric single arc technique (RapidArc), dynamic intensity-modulated technique and 3D conformal technique. Radiother. Oncol. J. Eur. Soc. Ther. Radiol. Oncol..

[46] Bush K., Townson R., Zavgorodni S. (2008). Monte Carlo simulation of RapidArc radiotherapy delivery. Phys. Med. Biol..

[47] Beltran C., Gray J., Merchant T.E. (2012). Intensity-modulated arc therapy for pediatric posterior fossa tumors. Int. J. Radiat. Oncol. Biol. Phys..

[48] Shaffer R., Nichol A.M., Vollans E., Fong M., Nakano S., Moiseenko V., Schmuland M., Ma R., McKenzie M., Otto K. (2010). A comparison of volumetric modulated arc therapy and conventional intensity-modulated radiotherapy for frontal and temporal high-grade gliomas. Int. J. Radiat. Oncol. Biol. Phys..

[49] Mohan R., Grosshans D. (2017). Proton therapy—Present and future. Pediatr. Blood Cancer.

[50] Ladra M.M., MacDonald S.M., Terezakis S.A. (2018). Proton therapy for central nervous system tumors in children. Pediatr. Blood Cancer.

[51] Chhabra A., Mahajan A. (2016). Treatment of common pediatric CNS malignancies with proton therapy. Chin. Clin. Oncol..

[52] Jimenez R.B., Sethi R., Depauw N., Pulsifer M.B., Adams J., McBride S.M., Ebb D., Fullerton B.C., Tarbell N.J., Yock T.I. (2013). Proton radiation therapy for pediatric medulloblastoma and supratentorial primitive neuroectodermal tumors: Outcomes for very young children treated with upfront chemotherapy. Int. J. Radiat. Oncol. Biol. Phys..

[53] St Clair W.H., Adams J.A., Bues M., Fullerton B.C., La Shell S., Kooy H.M., Loeffler J.S., Tarbell N.J. (2004). Advantage of protons compared to conventional X-ray or IMRT in the treatment of a pediatric patient with medulloblastoma. Int. J. Radiat. Oncol. Biol. Phys..

[54] Boehling N.S., Grosshans D.R., Bluett J.B., Palmer M.T., Song X., Amos R.A., Sahoo N., Meyer J.J., Mahajan A., Woo S.Y. (2012). Dosimetric comparison of three-dimensional conformal proton radiotherapy, intensity-modulated proton therapy, and intensity-modulated radiotherapy for treatment of pediatric craniopharyngiomas. Int. J. Radiat. Oncol. Biol. Phys..

[55] Beltran C., Roca M., Merchant T.E. (2012). On the benefits and risks of proton therapy in pediatric craniopharyngioma. Int. J. Radiat. Oncol. Biol. Phys..

[56] MacDonald S.M., Safai S., Trofimov A., Wolfgang J., Fullerton B., Yeap B.Y., Bortfeld T., Tarbell N.J., Yock T. (2008). Proton radiotherapy for childhood ependymoma: Initial clinical outcomes and dose comparisons. Int. J. Radiat. Oncol. Biol. Phys..

[57] Brower J.V., Gans S., Hartsell W.F., Goldman S., Fangusaro J.R., Patel N., Lulla R.R., Smiley N.P., Chang J.H., Gondi V. (2015). Proton therapy and helical tomotherapy result in reduced dose deposition to the pancreas in the setting of cranio-spinal irradiation for medulloblastoma: Implications for reduced risk of diabetes mellitus in long-term survivors. Acta Oncol..

[58] Eaton B.R., Esiashvili N., Kim S., Patterson B., Weyman E.A., Thornton L.T., Mazewski C., MacDonald T.J., Ebb D., MacDonald S.M. (2016). Endocrine outcomes with proton and photon radiotherapy for standard risk medulloblastoma. Neuro-Oncology.

[59] Wolden S.L. (2013). Protons for craniospinal radiation: Are clinical data important?. Int. J. Radiat. Oncol. Biol. Phys..

[60] Johnstone P.A., McMullen K.P., Buchsbaum J.C., Douglas J.G., Helft P. (2013). Pediatric CSI: Are protons the only ethical approach?. Int. J. Radiat. Oncol. Biol. Phys..

[61] Ladra M.M., Szymonifka J.D., Mahajan A., Friedmann A.M., Yong Yeap B., Goebel C.P., MacDonald S.M., Grosshans D.R., Rodriguez-Galindo C., Marcus K.J. (2014). Preliminary results of a phase II trial of proton radiotherapy for pediatric rhabdomyosarcoma. J. Clin. Oncol..

[62] Bishop A.J., Greenfield B., Mahajan A., Paulino A.C., Okcu M.F., Allen P.K., Chintagumpala M., Kahalley L.S., McAleer M.F., McGovern S.L. (2014). Proton beam therapy versus conformal photon radiation therapy for childhood craniopharyngioma: Multi-institutional analysis of outcomes, cyst dynamics, and toxicity. Int. J. Radiat. Oncol. Biol. Phys..

[63] McGovern S.L., Okcu M.F., Munsell M.F., Kumbalasseriyil N., Grosshans D.R., McAleer M.F., Chintagumpala M., Khatua S., Mahajan A. (2014). Outcomes and acute toxicities of proton therapy for pediatric atypical teratoid/rhabdoid tumor of the central nervous system. Int. J. Radiat. Oncol. Biol. Phys..

[64] Sethi R.V., Giantsoudi D., Raiford M., Malhi I., Niemierko A., Rapalino O., Caruso P., Yock T.I., Tarbell N.J., Paganetti H. (2014). Patterns of failure after proton therapy in medulloblastoma; linear energy transfer distributions and relative biological effectiveness associations for relapses. Int. J. Radiat. Oncol. Biol. Phys..

[65] Sato M., Gunther J.R., Mahajan A., Jo E., Paulino A.C., Adesina A.M., Jones J.Y., Ketonen L.M., Su J.M., Okcu M.F. (2017). Progression-free survival of children with localized ependymoma treated with intensity-modulated radiation therapy or proton-beam radiation therapy. Cancer.

[66] Gunther J.R., Sato M., Chintagumpala M., Ketonen L., Jones J.Y., Allen P.K., Paulino A.C., Okcu M.F., Su J.M., Weinberg J. (2015). Imaging Changes in Pediatric Intracranial Ependymoma Patients Treated With Proton Beam Radiation Therapy Compared to Intensity Modulated Radiation Therapy. Int. J. Radiat. Oncol. Biol. Phys..

[67] Sethi R.V., Shih H.A., Yeap B.Y., Mouw K.W., Petersen R., Kim D.Y., Munzenrider J.E., Grabowski E., Rodriguez-Galindo C., Yock T.I. (2014). Second nonocular tumors among survivors of retinoblastoma treated with contemporary photon and proton radiotherapy. Cancer.

[68] Chung C.S., Yock T.I., Nelson K., Xu Y., Keating N.L., Tarbell N.J. (2013). Incidence of second malignancies among patients treated with proton versus photon radiation. Int. J. Radiat. Oncol. Biol. Phys..

[69] Geng C., Moteabbed M., Xie Y., Schuemann J., Yock T., Paganetti H. (2016). Assessing the radiation-induced second cancer risk in proton therapy for pediatric brain tumors: The impact of employing a patient-specific aperture in pencil beam scanning. Phys. Med. Biol..

[70] Brenner D.J., Hall E.J. (2008). Secondary neutrons in clinical proton radiotherapy: A charged issue. Radiother. Oncol. J. Eur. Soc. Ther. Radiol. Oncol..

[71] Moteabbed M., Yock T.I., Paganetti H. (2014). The risk of radiation-induced second cancers in the high to medium dose region: A comparison between passive and scanned proton therapy, IMRT and VMAT for pediatric patients with brain tumors. Phys. Med. Biol..

[72] Lomax A. (1999). Intensity modulation methods for proton radiotherapy. Phys. Med. Biol..

[73] Lomax A.J., Boehringer T., Coray A., Egger E., Goitein G., Grossmann M., Juelke P., Lin S., Pedroni E., Rohrer B. (2001). Intensity modulated proton therapy: A clinical example. Med. Phy..

[74] Frank S.J., Cox J.D., Gillin M., Mohan R., Garden A.S., Rosenthal D.I., Gunn G.B., Weber R.S., Kies M.S., Lewin J.S. (2014). Multifield optimization intensity modulated proton therapy for head and neck tumors: A translation to practice. Int. J. Radiat. Oncol. Biol. Phys..

[75] Holliday E.B., Kocak-Uzel E., Feng L., Thaker N.G., Blanchard P., Rosenthal D.I., Gunn G.B., Garden A.S., Frank S.J. (2016). Dosimetric advantages of intensity-modulated proton therapy for oropharyngeal cancer compared with intensity-modulated radiation: A case-matched control analysis. Med. Dosim..

[76] Sio T.T., Lin H.K., Shi Q., Gunn G.B., Cleeland C.S., Lee J.J., Hernandez M., Blanchard P., Thaker N.G., Phan J. (2016). Intensity Modulated Proton Therapy Versus Intensity Modulated Photon Radiation Therapy for Oropharyngeal Cancer: First Comparative Results of Patient-Reported Outcomes. Int. J. Radiat. Oncol. Biol. Phys..

[77] Haas-Kogan D., Indelicato D., Paganetti H., Esiashvili N., Mahajan A., Yock T., Flampouri S., MacDonald S., Fouladi M., Stephen K. (2018). National Cancer Institute Workshop on Proton Therapy for Children: Considerations Regarding Brainstem Injury. Int. J. Radiat. Oncol. Biol. Phys..

[78] Ebner D.K., Kamada T. (2016). The Emerging Role of Carbon-Ion Radiotherapy. Front. Oncol..

[79] Mohamad O., Imai R., Kamada T., Nitta Y., Araki N. (2018). Carbon ion radiotherapy for inoperable pediatric osteosarcoma. Oncotarget.

[80] Blattmann C., Oertel S., Schulz-Ertner D., Rieken S., Haufe S., Ewerbeck V., Unterberg A., Karapanagiotou-Schenkel I., Combs S.E., Nikoghosyan A. (2010). Non-randomized therapy trial to determine the safety and efficacy of heavy ion radiotherapy in patients with non-resectable osteosarcoma. BMC Cancer.

[81] Combs S.E., Nikoghosyan A., Jaekel O., Karger C.P., Haberer T., Munter M.W., Huber P.E., Debus J., Schulz-Ertner D. (2009). Carbon ion radiotherapy for pediatric patients and young adults treated for tumors of the skull base. Cancer.

[82] Aggarwal R., Yeung D., Kumar P., Muhlbauer M., Kun L.E. (1997). Efficacy and feasibility of stereotactic radiosurgery in the primary management of unfavorable pediatric ependymoma. Radiother. Oncol. J. Eur. Soc. Ther. Radiol. Oncol..

[83] Hodgson D.C., Goumnerova L.C., Loeffler J.S., Dutton S., Black P.M., Alexander E., Xu R., Kooy H., Silver B., Tarbell N.J. (2001). Radiosurgery in the management of pediatric brain tumors. Int. J. Radiat. Oncol. Biol. Phys..

[84] Marcus K.J., Goumnerova L., Billett A.L., Lavally B., Scott R.M., Bishop K., Xu R., Young Poussaint T., Kieran M., Kooy H. (2005). Stereotactic radiotherapy for localized low-grade gliomas in children: Final results of a prospective trial. Int. J. Radiat. Oncol. Biol. Phys..

[85] Weintraub D., Yen C.P., Xu Z., Savage J., Williams B., Sheehan J. (2012). Gamma knife surgery of pediatric gliomas. J. Neurosurg. Pediatr..

[86] Barcia J.A., Barcia-Salorio J.L., Ferrer C., Ferrer E., Algas R., Hernandez G. (1994). Stereotactic radiosurgery of deeply seated low grade gliomas. Acta Neurochir. Suppl..

[87] Abe M., Tokumaru S., Tabuchi K., Kida Y., Takagi M., Imamura J. (2006). Stereotactic radiation therapy with chemotherapy in the management of recurrent medulloblastomas. Pediatr. Neurosurg..

[88] Patrice S.J., Tarbell N.J., Goumnerova L.C., Shrieve D.C., Black P.M., Loeffler J.S. (1995). Results of radiosurgery in the management of recurrent and residual medulloblastoma. Pediatr. Neurosurg..

[89] Barua K.K., Ehara K., Kohmura E., Tamaki N. (2003). Treatment of recurrent craniopharyngiomas. Kobe J. Med. Sci..

[90] Jeon C., Kim S., Shin H.J., Nam D.H., Lee J.I., Park K., Kim J.H., Jeon B., Kong D.S. (2011). The therapeutic efficacy of fractionated radiotherapy and gamma-knife radiosurgery for craniopharyngiomas. J. Clin. Neurosci..

[91] Niranjan A., Kano H., Mathieu D., Kondziolka D., Flickinger J.C., Lunsford L.D. (2010). Radiosurgery for craniopharyngioma. Int. J. Radiat. Oncol. Biol. Phys..

[92] Xu Z., Yen C.P., Schlesinger D., Sheehan J. (2011). Outcomes of Gamma Knife surgery for craniopharyngiomas. J. Neuro-Oncol..

[93] Foy A.B., Wetjen N., Pollock B.E. (2010). Stereotactic radiosurgery for pediatric arteriovenous malformations. Neurosurg. Clin. N. Am..

[94] Hong J.C., Salama J.K. (2017). The expanding role of stereotactic body radiation therapy in oligometastatic solid tumors: What do we know and where are we going?. Cancer Treat. Rev..

[95] Kim H., Rajagopalan M.S., Beriwal S., Huq M.S., Smith K.J. (2015). Cost-effectiveness analysis of single fraction of stereotactic body radiation therapy compared with single fraction of external beam radiation therapy for palliation of vertebral bone metastases. Int. J. Radiat. Oncol. Biol. Phys..

[96] Rao A.D., Rashid A.S., Chen Q., Villar R.C., Kobyzeva D., Nilsson K., Dieckmann K., Nechesnyuk A., Ermoian R., Alcorn S. (2017). Reirradiation for Recurrent Pediatric Central Nervous System Malignancies: A Multi-institutional Review. Int. J. Radiat. Oncol. Biol. Phys..

[97] Massimino M., Miceli R., Giangaspero F., Boschetti L., Modena P., Antonelli M., Ferroli P., Bertin D., Pecori E., Valentini L. (2016). Final results of the second prospective AIEOP protocol for pediatric intracranial ependymoma. Neuro-Oncology.

[98] Alcorn S.R., Chen M.J., Claude L., Dieckmann K., Ermoian R.P., Ford E.C., Malet C., MacDonald S.M., Nechesnyuk A.V., Nilsson K. (2014). Practice patterns of photon and proton pediatric image guided radiation treatment: Results from an International Pediatric Research consortium. Pract. Radiat. Oncol..

[99] Hess C.B., Thompson H.M., Benedict S.H., Seibert J.A., Wong K., Vaughan A.T., Chen A.M. (2016). Exposure Risks Among Children Undergoing Radiation Therapy: Considerations in the Era of Image Guided Radiation Therapy. Int. J. Radiat. Oncol. Biol. Phys..

[100] Beltran C., Krasin M.J., Merchant T.E. (2011). Inter- and intrafractional positional uncertainties in pediatric radiotherapy patients with brain and head and neck tumors. Int. J. Radiat. Oncol. Biol. Phys..

[101] Murphy M.J., Balter J., Balter S., BenComo J.A., Das I.J., Jiang S.B., Ma C.M., Olivera G.H., Rodebaugh R.F., Ruchala K.J. (2007). The management of imaging dose during image-guided radiotherapy: Report of the AAPM Task Group 75. Med. Phys..

[102] Kornguth D., Mahajan A., Frija E., Chang E., Pelloski C., Woo S. (2006). 2091: Shape Variability of Craniopharyngioma as Measured on CT-on-Rails During Radiotherapy Treatment. Int. J. Radiat. Oncol. Biol. Phys..

[103] Winkfield K.M., Linsenmeier C., Yock T.I., Grant P.E., Yeap B.Y., Butler W.E., Tarbell N.J. (2009). Surveillance of Craniopharyngioma Cyst Growth in Children Treated With Proton Radiotherapy. Int. J. Radiat. Oncol. Biol. Phys..

[104] Beltran C., Naik M., Merchant T.E. (2010). Dosimetric effect of target expansion and setup uncertainty during radiation therapy in pediatric craniopharyngioma. Radiother. Oncol. J. Eur. Soc. Ther. Radiol. Oncol..

[105] Fukunaga-Johnson N., Lee J.H., Sandler H.M., Robertson P., McNeil E., Goldwein J.W. (1998). Patterns of failure following treatment for medulloblastoma: Is it necessary to treat the entire posterior fossa?. Int. J. Radiat. Oncol. Biol. Phys..

[106] Wolden S.L., Dunkel I.J., Souweidane M.M., Happersett L., Khakoo Y., Schupak K., Lyden D., Leibel S.A. (2003). Patterns of failure using a conformal radiation therapy tumor bed boost for medulloblastoma. J. Clin. Oncol..

[107] Michalski J.M., Janss A., Vezina G., Gajjar A., Pollack I., Merchant T.E., FitzGerald T.J., Booth T., Tarbell N.J., Li Y. (2016). Results of COG ACNS0331: A Phase III Trial of Involved-Field Radiotherapy (IFRT) and Low Dose Craniospinal Irradiation (LD-CSI) with Chemotherapy in Average-Risk Medulloblastoma: A Report from the Children’s Oncology Group. Int. J. Radiat. Oncol. Biol. Phys..

[108] Alapetite C., Brisse H., Patte C., Raquin M.A., Gaboriaud G., Carrie C., Habrand J.L., Thiesse P., Cuilliere J.C., Bernier V. (2010). Pattern of relapse and outcome of non-metastatic germinoma patients treated with chemotherapy and limited field radiation: The SFOP experience. Neuro-Oncology.

[109] Calaminus G., Kortmann R., Worch J., Nicholson J.C., Alapetite C., Garre M.L., Patte C., Ricardi U., Saran F., Frappaz D. (2013). SIOP CNS GCT 96: Final report of outcome of a prospective, multinational nonrandomized trial for children and adults with intracranial germinoma, comparing craniospinal irradiation alone with chemotherapy followed by focal primary site irradiation for patients with localized disease. Neuro-Oncology.

[110] Rogers S.J., Mosleh-Shirazi M.A., Saran F.H. (2005). Radiotherapy of localised intracranial germinoma: Time to sever historical ties?. Lancet Oncol..

[111] Khatua S., Fangusaro J., Dhall G., Boyett J., Wu S., Bartels U. (2016). GC-17THE CHILDREN’S ONCOLOGY GROUP (COG) CURRENT TREATMENT APPROACH FOR CHILDREN WITH NEWLY DIAGNOSED CENTRAL NERVOUS SYSTEM (CNS) LOCALIZED GERMINOMA (ACNS1123 STRATUM 2). Neuro-Oncology.

[112] Goldman S., Bouffet E., Fisher P.G., Allen J.C., Robertson P.L., Chuba P.J., Donahue B., Kretschmar C.S., Zhou T., Buxton A.B. (2015). Phase II Trial Assessing the Ability of Neoadjuvant Chemotherapy With or Without Second-Look Surgery to Eliminate Measurable Disease for Nongerminomatous Germ Cell Tumors: A Children’s Oncology Group Study. J. Clin. Oncol..

[113] Cahlon O., Dunkel I., Gilheeney S., Khakoo Y., Souweidane M., De Braganca K., Kramer K., Wolden S. (2014). Craniospinal Radiation Therapy May Not Be Necessary for Localized Nongerminomatous Germ Cell Tumors (NGGCT). Int. J. Radiat. Oncol. Biol. Phys..

[114] Packer R.J., Goldwein J., Nicholson H.S., Vezina L.G., Allen J.C., Ris M.D., Muraszko K., Rorke L.B., Wara W.M., Cohen B.H. (1999). Treatment of children with medulloblastomas with reduced-dose craniospinal radiation therapy and adjuvant chemotherapy: A Children’s Cancer Group Study. J. Clin. Oncol..

[115] Goldwein J.W., Radcliffe J., Johnson J., Moshang T., Packer R.J., Sutton L.N., Rorke L.B., D’Angio G.J. (1996). Updated results of a pilot study of low dose craniospinal irradiation plus chemotherapy for children under five with cerebellar primitive neuroectodermal tumors (medulloblastoma). Int. J. Radiat. Oncol. Biol. Phys..

[116] Ramaswamy V., Remke M., Bouffet E., Bailey S., Clifford S.C., Doz F., Kool M., Dufour C., Vassal G., Milde T. (2016). Risk stratification of childhood medulloblastoma in the molecular era: The current consensus. Acta Neuropathol..

